# Watt-scale super-octave mid-infrared intrapulse difference frequency generation

**DOI:** 10.1038/s41377-018-0099-5

**Published:** 2018-11-28

**Authors:** Christian Gaida, Martin Gebhardt, Tobias Heuermann, Fabian Stutzki, Cesar Jauregui, Jose Antonio-Lopez, Axel Schülzgen, Rodrigo Amezcua-Correa, Andreas Tünnermann, Ioachim Pupeza, Jens Limpert

**Affiliations:** 1 Institute of Applied Physics, Abbe Center of Photonics, Friedrich-Schiller-Universität Jena, Albert-Einstein-Str. 15, 07745 Jena, Germany; 2 Helmholtz-Institute Jena, Fröbelstieg 3, 07743 Jena, Germany; 3 Fraunhofer Institute for Applied Optics and Precision Engineering, Albert-Einstein-Str. 7, 07745 Jena, Germany; 4 CREOL, College of Optics and Photonics, University of Central Florida, Orlando, FL 32816 USA; 5 Max-Planck-Institute of Quantum Optics, Hans-Kopfermann-Str. 1, 85748 Garching, Germany

## Abstract

The development of high-power, broadband sources of coherent mid-infrared radiation is currently the subject of intense research that is driven by a substantial number of existing and continuously emerging applications in medical diagnostics, spectroscopy, microscopy, and fundamental science. One of the major, long-standing challenges in improving the performance of these applications has been the construction of compact, broadband mid-infrared radiation sources, which unify the properties of high brightness and spatial and temporal coherence. Due to the lack of such radiation sources, several emerging applications can be addressed only with infrared (IR)-beamlines in large-scale synchrotron facilities, which are limited regarding user access and only partially fulfill these properties. Here, we present a table-top, broadband, coherent mid-infrared light source that provides brightness at an unprecedented level that supersedes that of synchrotrons in the wavelength range between 3.7 and 18 µm by several orders of magnitude. This result is enabled by a high-power, few-cycle Tm-doped fiber laser system, which is employed as a pump at 1.9 µm wavelength for intrapulse difference frequency generation (IPDFG). IPDFG intrinsically ensures the formation of carrier-envelope-phase stable pulses, which provide ideal prerequisites for state-of-the-art spectroscopy and microscopy.

## Mid-infrared photonics: bright broadband source

A table-top-sized, coherent light source that offers a compact and bright alternative to a synchrotron in the 4−18 µm spectral range has been developed by a German-US research team. The team used a novel ultrashort (16 fs) pulse, high power Tm-doped fiber laser operating at 1.9 µm to induce a nonlinear frequency downconversion process called intrapulse difference frequency generation in a crystal of GaSe. The broad spectral coverage and high brightness render this mid-infrared source a unique tool for state-of-the art spectroscopy and microscopy. The team says that the compactness and simplicity of the presented approach brings exciting prospects for the future accessibility, in particular for emerging applications that are currently addressed only with mid-infrared beamlines in large-scale synchrotron facilities.

## Introduction

The energies of fundamental modes of atomic vibrations in molecules correspond to mid-infrared (mid-IR) frequencies in the range between several hundreds and several thousands of inverse centimeters, which are often referred to as the molecular fingerprint region (usually located in the wavenumber range between 500 and 5000 cm^−1^ or wavelengths between 2 and 20 µm). Molecular vibrations that exhibit electric dipole moments can be readily accessed by linear absorption spectroscopy, which causes sample-specific, spectral absorption “fingerprints” that are abundant in information about the molecular composition, structure, and conformation^1–5^. For more than half a century, the powerful technique of Fourier transform IR spectroscopy (FTIR)^1^ has enabled a plethora of applications in fields that range from analytical chemistry^1,2^ to environmental monitoring^1^ to life sciences^3,4^. Owing to the lack of viable alternatives and their unparalleled compactness and cost-effectiveness, thermal radiation sources are employed in the vast majority of absorption spectrometers for the mid-IR range^1^. However, the spatially incoherent nature of their radiation imposes a stringent trade-off between the usable IR power and the propagation distance through the sample (and through the FTIR interferometer), which causes severe limitations of the achievable signal-to-noise ratio and/or spectral resolution. This disadvantage can be overcome by IR emission of synchrotrons, which—owing to their spatial coherence—attain considerably higher brightness levels^6^. For FTIR measurements, this result enables higher sensitivity and the implementation of extensions, such as the combination of spectroscopy with microscopy^4^. However, synchrotrons are building-size facilities, which severely restricts their usability.

During the past two decades, ultrafast laser technology has undergone rapid development that has spawned novel radiation sources and advanced spectroscopy techniques that harness their outstanding spatial and temporal coherence properties. For instance, THz time-domain spectroscopy^7^, which directly measures the complete electric field of ultrashort far-infrared pulses, has practically replaced FTIR in the region of frequencies to a few THz. Another example is precision spectroscopy, which employs frequency combs^5^. With appropriate detection schemes, such as the dual-comb configuration^8^, or FTIR with an optical delay path matched to the distance between the comb pulses^9^, frequency combs enable FTIR-like measurements with frequency resolutions to the comb linewidth. Applying these techniques to the previously mentioned field of applications in the mid-IR is promising for unprecedented regimes of sensitivity, spectral resolution, and acquisition time.

**Fig. 1:**
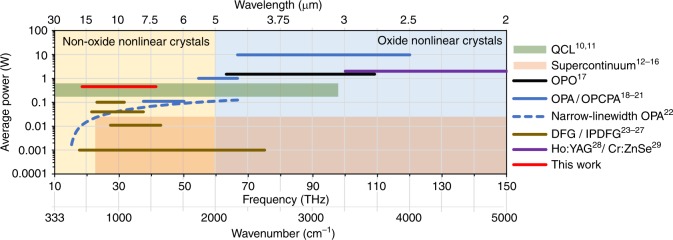
State-of-the-art mid-IR sources^10^. Only a few concepts, such as quantum-cascade lasers^11, 12^ (QCL, green area) or CO_2_ lasers^13^, provide direct mid-IR generation. However, these sources emit rather narrow spectra. Supercontinuum generation (supercontinuum, orange area) in nonlinear fibers^14–16^, waveguides^17^ and crystals^13^ represent extensively explored routes towards broad mid-IR spectra that are limited to average powers between 10 µW and 50 mW. Frequency downconversion in nonlinear crystals is a prominent alternative technique to obtain high power mid-IR emission. Operating in the transparency window for oxide nonlinear crystals (blue-colored background), optical parametric oscillators (OPO, black line)^18^ have attained watt-level and optical parametric amplifiers (OPA, blue lines)^19, 20^ and have attained 10 Watt-level average output power at a wavelength of up to 5 µm. OPAs and OPCPAs with longer wavelength emission have been realized by utilizing the transparency properties of nonoxide nonlinear crystals (light orange colored background), which provide an extended transparency window^21, 22^. Steinle et al. demonstrated the advantage of a long wavelength driver in conjunction with nonoxide nonlinear crystals with a narrow-linewidth, cascaded OPA (narrow-linewidth OPA, dashed blue line) that attain average powers in the 1−100 mW range and tunability between 4.5 and 20 µm^23^. Direct pumping at longer wavelengths for broadband difference frequency generation (DFG, brown line) or intrapulse DFG (IPDFG, brown line), however, has so far been relatively unattractive because the used pump lasers either had poor average-power scalability^24, 25^ or could only attain high pulse energies at the expense of relatively low repetition rates in the kHz range^26^. Utilizing a short wavelength driver for IPDFG in a nonoxide nonlinear crystal has resulted in a 100 mW average power broadband frequency comb that is spectrally centered at 11.5 µm^27^. In addition to the ongoing development of high average power Ho:YAG thin-disk oscillators^28^ and mid-IR Cr:ZnSe-bulk lasers^29^ (Ho:YAG / Cr:ZnS, violet line), the results presented in this work (red line) are based on the recent development of high-power Tm-doped fiber laser emitting few-cycle pulses that are centered at 1.9 µm wavelength^30^ as a driving source for IPDFG

A laser-based source that covers the entire molecular fingerprint region and combines the key properties desirable for coherent spectroscopy does not exist: broad bandwidth (preferably without the need for tuning), spatial and temporal coherence, high repetition rate and high average power. In the absence of broadband mid-IR laser gain media, coherent broadband mid-IR sources usually employ parametric downconversion of ultrashort laser pulses generated by matured visible/near-IR laser technologies^10–30^. Figure 1 shows a selection of these sources, which are representative of the state of the art. These sources typically employ oxide materials as nonlinear media, which are not transparent for longer wavelengths (Fig. 1). Nonoxide crystals provide excellent transmission in the wavelength region, which ranges from 5 to 20 µm (represented by light orange background color in Fig. 1); however, few crystals have low absorption at the 1 µm wavelength, where most high-power ultrafast lasers operate^27,31^. Among the different parametric mid-IR sources, intrapulse difference frequency generation (IPDFG)^24,25,27,32^ uniquely combines compactness, intrinsic carrier-envelope phase (CEP) stability^33^ and short pulse durations, which are ideal prerequisites for frequency comb spectroscopy and field-resolved spectroscopy via electro-optical sampling. However, driving lasers at 1 µm exhibit two drawbacks. First, the relatively low nonlinear coefficient of LiGaS2 (LGS)—the only crystal that is suitable for multi-10 W IPDFG driven at 1 μm^27,31^—produces a conversion efficiency of only 0.2% prior to the onset of multiphoton absorption. Second, phase matching restricts the bandwidth of the highest-efficiency conversion to a few micrometers around the central wavelength.

In this study, we demonstrate that both of these limitations can be substantially alleviated with a compact source of few-cycle 1.9 μm pulses and a suitable IPDFG crystal. Due to the lower photon energy of the driving pulses, nonoxide crystals with a significantly higher nonlinearity, such as GaSe, can be used^31^. In addition, improved phase matching conditions and broadband transmission enable the efficient generation of a broader mid-IR spectrum^26^. Compared with IPDFG with a 1 µm driving laser^27^, we demonstrate an increase in conversion efficiency by one order of magnitude accompanied by a threefold increase in mid-IR bandwidth (at −10 dB intensity). The table-top mid-IR source presented in this study supersedes the brightness of synchrotron radiation by several orders of magnitude over more than one octave of bandwidth while offering the benefit of intrinsic phase stability.

## Materials and methods

### Few-cycle thulium-doped fiber laser

**Fig. 2:**
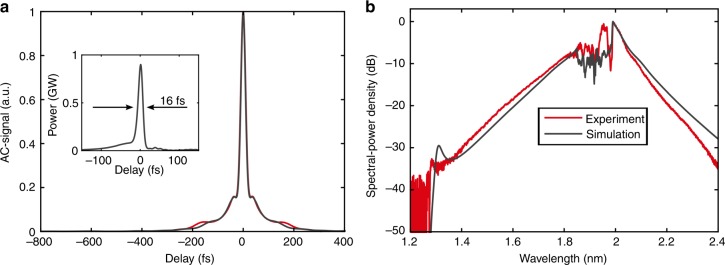
Output characteristics of the few-cycle thulium-doped fiber laser for intrapulse difference frequency generation (IPDFG). **a** Measured (red) and simulated (black) intensity autocorrelation of the nonlinear compressed pulses. The inset shows the numerically retrieved intensity envelope of the pulse with a peak power of 0.9 GW. **b** Measured spectrum (red) and simulated (black) spectrum of the nonlinearly compressed pulses emitted by the Tm:FCPA-seeded antiresonant hollow core fiber at 1.25 MHz repetition rate

A thulium-doped fiber chirped-pulse amplification system (Tm:FCPA) with an architecture that is comparable to that described in ref. ^34^ served as the laser source for a subsequent nonlinear self-compression stage^30^ and intrapulse DFG. In contrast to the result in ref. ^33^, the Tm:FCPA was operated with larger spectral bandwidth centered at 1920 nm and delivered at 110 fs pulses (intensity FWHM duration). In addition, detrimental propagation effects, such as thermal blooming and pulse quality degradation from the absorption of atmospheric water vapor at wavelengths <1930 nm, were efficiently mitigated by placing high-power sections of the laser in vacuum^35^. The Tm:FCPA in this experiment delivered 31.4 W of average power, which corresponds to 24 µJ of pulse energy at a repetition rate of 1.25 MHz. These pulses were nonlinearly compressed in an antiresonant hollow-core fiber (ARHCF) with a length of 55 cm and a core diameter of 51 µm. The input side of the ARHCF was held at 0.4 bar helium to reduce the effects of molecular water absorption while providing sufficient convection cooling of the fiber tip for long-term operation. The fiber output side was held at 3 bar argon as nonlinear medium, which causes a pressure gradient in the hollow core and significant spectral broadening of the pulses from the Tm:FCPA due to self-phase modulation upon propagation along the ARHCF. Simultaneously, the pulses were compressed due to the anomalous dispersion of the ARHCF in this wavelength region. The output of the ARHCF was characterized by measuring the intensity autocorrelation (Fig. 2a) and the nonlinearly broadened spectrum (Fig. 2b). In addition, numerical modeling of the nonlinear self-compression stage was performed by solving the generalized nonlinear Schrödinger equation, including the ionization level of the helium gas and the transmission properties and dispersion of the ARHCF^30^. The numerical results match the experimental features in the temporal and spectral domain, which reveals a pulse duration of 16 fs and a peak power of 0.9 GW (inset of Fig. 2a).

### Intrapulse difference frequency generation in GaSe

**Fig. 3:**
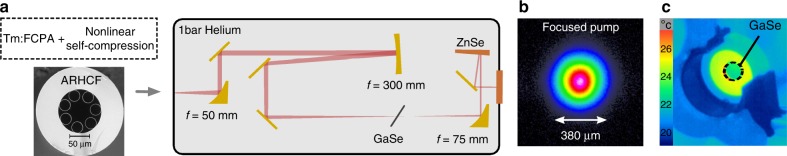
Schematic experimental setup for intrapulse difference frequency generation. **a** The experimental setup for intrapulse DFG comprises a table-top thulium-doped fiber chirped-pulse amplification system (Tm:FCPA), an antiresonant hollow-core-fiber-based nonlinear self-compression stage and a separate chamber filled with 1 bar of helium gas for the mitigation of thermal blooming^35^, which enables efficient intrapulse difference frequency generation. **b** Beam focus with a 380 µm 1/e² width, which produces a peak intensity of 1.59 TW/cm² at the GaSe crystal for intrapulse DFG. **c** Thermal camera image of the GaSe crystal, while illuminating with an 80 W-average-power laser for several minutes

The IPDFG setup is schematically shown in Fig. 3a. The self-compressed pulses from the ARHCF were collimated by an *f* = 50 mm parabola that was directed to a separate chamber filled with 1 bar helium and focused on a 1-mm-thick GaSe nonlinear crystal with an *f* = 300 mm spherical mirror. This step produced a beam focus of 380 µm 1/e^2^ width (Fig. 3b), which generated a peak intensity of 1.59 TW/cm^2^ at the input facet of the GaSe crystal, which is located immediately below the experimentally determined damage threshold of 1.7 TW/cm^2^. The generated idler and the remaining pump and signal were collimated by a 75 mm parabola. A ZnSe-wedge was used to reduce the total average power before the beam was directed out of the chamber. In this manner, thermal blooming due to molecular water absorption in air was efficiently mitigated, which enabled characterization of the generated idler with a Fourier transform interferometer, a thermal power meter and a mid-IR beam profiler (detection range 2−20 µm). The remaining pump light was suppressed by a longpass filter (>40 dB suppression at 2 µm wavelength). The losses due to the ZnSe-wedge, ZnSe-output window and longpass filters have been determined at a low repetition rate, which enables accurate correction of the measured average power (refer to supplementary information). The high average power handling capability of this GaSe crystal has been separately tested with a broadband amplified spontaneous emission source that provides 80 W of average power (>100 nm width at −10 dB centered at 1900 nm). An absorption-induced thermal gradient has not been observed (Fig. 3c), which corroborates the power-scaling potential of IPDFG in GaSe.

### Phase matching considerations

**Fig. 4:**
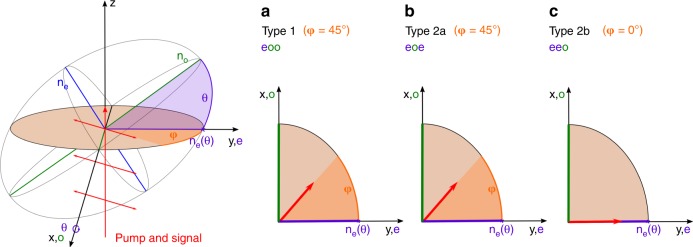
Phase matching types. Crystal orientation and polarization states of the input pulses (pump and signal) for type 1 (**a**), type 2a (**b**), and type 2b (**c**) phase matching are shown for intrapulse difference frequency generation in GaSe. Type 1 and type 2a phase matching are typically achieved with input pulses that are linearly polarized with *φ* = 45° orientation to the *y*-axes in the *y*−*x* plane, which causes the splitting of a pump and signal to the projected fast (*e*) and slow (*o*) axes of the crystal. This result enables generation of the idler wave in the projected *e*-or *o*-axes of the crystal (*eoe* or *eoo*). Type 2b phase matching requires *p*-polarized input light that corresponds to *φ* = 0°, with a pump and signal propagating in the projected *e*-axes of the crystal and generation of the idler wave in the *o*-axes of the crystal (*eeo*)

**Fig. 5:**
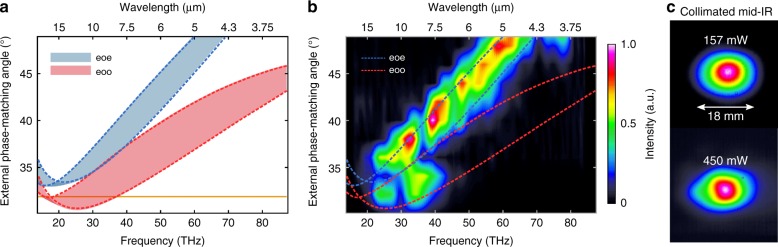
Phase- matching calculations and experiment. **a** The phase matching calculation produces two possible branches with respect to the external angle of incidence on the GaSe-crystal and generated idler frequencies of ordinary (type 1, *eoo*) or extraordinary (type 2a, *eoe*) polarization. The width of each branch represents phase matched scenarios (Δ*k* = 0) for intrapulse difference frequency generation (IPDFG), which considers pump waves that range from 1300 to 2300 nm (always *e*-polarized) and signal waves of up to 2400 nm (always *o*-polarized) of the few-cycle input pulse (see spectrum in Fig. 2b). The orange line indicates the external angle of incidence that was chosen for the high-power experiment. **b** Generated mid-IR spectra for a sweep of the external angle of incidence on the GaSe crystal. The blue dashed lines and red dashed lines represent the calculations for *eoe* phase matching and *eoo* phase matching, respectively. The orange line indicates the external angle of incidence that was chosen for the high-power experiment presented in this paper. **c** Typical collimated mid-IR beam profiles at a 400 kHz pulse repetition rate/157 mW average power and a 1.25 MHz pulse repetition rate/450 mW average power. The weak beam deformation at the highest power is caused by thermal deformation of the collimating gold parabola

Efficient mid-IR generation via IPDFG requires phase matching of the pump, signal and idler waves, which can be achieved by exploiting the crystal birefringence, whereas the synchronization of the signal and pump is intrinsically ensured for transform-limited input pulses. The dispersion relations of the ordinary and extraordinary GaSe crystal axes were estimated with Sellmeier coefficients provided by the crystal manufacturer (refer to supplementary information). Based on the spectral band of the few-cycle pulses launched at the crystal, the phase mismatch (∆*k* = *k*_p_ − *k*_s_ − *k*_i_) can be calculated for the pump (*k*_p_), signal (*k*_s_), and idler (*k*_i_) wave vectors. Since GaSe is only available in a Z-cut orientation, the condition Δ*k* = 0 and the targeted idler wavelengths in the molecular fingerprint region can theoretically be fulfilled in three different configurations, which are schematically illustrated in Fig. 4. Simultaneous propagation of signal and pump waves in the projected extraordinary axis (e) and idler waves in the ordinary axis (*eeo*, phase matching type 2b) implies large external angles of incidence (*θ*), and consequently, large idler output angles, which cause total internal reflection of the generated mid-IR at the output side of the GaSe crystal. Phase matching and efficient mid-IR idler output can only be achieved with propagation of the pump waves in the projected *e*-axis, the signal waves in the *o*-axis and the idler waves in the *e*- or *o*-axis (type 2a, *eoe* or type 1, *eoo*). The two possible branches for phase matching and sufficiently small external angles of incidence (*θ*) vs. idler frequency are illustrated in Fig. 5a. The width of each branch is attributed to sweeping the available pump wavelength from 1300 to 2300 nm using the remaining spectrum to the 2400 nm wavelength as a signal and fulfilling the condition Δ*k* = 0.

## Results and discussion

### Broadband tuning in the mid-IR

For type I phase matching (*eoe*) the crystal orientation and input polarization were optimized to follow the scheme shown in Fig. 4a. We tuned the crystal between *θ* = 29° and *θ* = 49° external angle of incidence (Fig. 5b) to optimize for efficient broadband mid-IR generation in the 3−20 µm wavelength range. This optimization was performed with a reduced pulse repetition rate of 400 kHz to maintain the average power at a level that was sufficiently low for operation in normal atmosphere, which enabled manual tuning of the GaSe crystal. For each external angle of incidence (*θ*), the idler average power was maximized by slightly tuning the orientation of the crystal (Δ*φ* < 1°) with respect to the input polarization. The generated idler was spectrally characterized with a Fourier transform interferometer that was equipped with a thermal sensor. The center frequency of the generated mid-IR light decreased for lower angles of incidence (*θ*) and fits the theoretically predicted *eoe*-branch (compare with Fig. 5a). The generated mid-IR average power at a pulse repetition rate of 400 kHz was 157 mW at an external input angle of 32°, as indicated by the orange lines in Fig. 5a, b; it did not significantly change for the entire tuning range. The beam profile at this average power level is depicted in Fig. 5c and shows no sign of beam quality degradation. The slight ellipticity can be attributed to spatial walk-off between idler and pump waves and/or small alignment errors of the collimating parabola. Angles of incidence larger than 49° were not possible due to beam clipping at the crystal holder, which prevented us from observing the high-frequency limit that was ultimately set by the input spectral bandwidth. Input angles smaller than 30° produced an abrupt reduction of the mid-IR average power, which indicates that no phase matching was achievable beyond this point. This observation is also consistent with the calculated phase matching conditions (Fig. 5a). The *eoe*-branch appears to be more efficient over a wide range of external angles of incidence. Note that experimental generation of idler frequencies that fit the *eoo*-branch for external angles smaller than 40° was possible by detuning the angle *φ* and changing the ratio of the pump and signal intensities. However, the average mid-IR output power was significantly reduced. When optimizing for the highest mid-IR average power, the *eoe*-branch was always more efficient for external angles of incidence larger than 35°. The *eoo*-branch becomes similarly efficient for external angles of incidence smaller than 34°. This behavior cannot be understood by looking at the phase matching conditions in Fig. 5a. The significantly higher efficiency of the *eoe*-branch compared with the *eoo*-branch is caused by a complex interplay of group-delay-dispersion mismatch and temporal chirp of the few-cycle input pulse during propagation in the extraordinary and ordinary crystal axes. This result produces a longer nonlinear interaction length of pump and signal waves that fulfill the *eoe*-phase matching conditions (Fig. 5b), which render the conversion process efficient, even for short idler wavelengths. The influences of group delay mismatch and temporal chirp on conversion efficiency are significantly reduced for pump and signal waves that are in close wavelength proximity, which renders generation of long idler wavelengths generally more efficient even in the case of *eoo*-phase matching.

### High brightness mid-IR generation

**Fig. 6:**
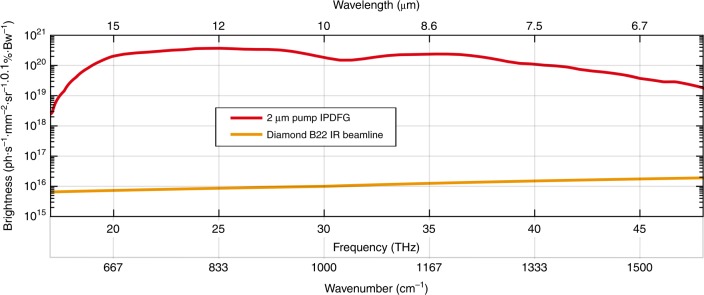
Brightness comparison of IPDPG and synchrotron radiation. High-power mid-IR spectra generated by intrapulse difference frequency generation, pumped at 2 µm wavelength (this work, red). The brightness of the high-power table-top mid-IR source in this work exceeds that of large-scale facility synchrotrons, e.g., the Diamond B22 IR beamline^6^, by 4 orders of magnitude in the 7.5–15 µm wavelength range

The external angle of incidence at 32° (refer to orange lines in Fig. 5a, b) was employed for experiments at higher average power. To avoid detrimental water vapor absorption effects in the atmosphere^35^ we evacuated the DFG chamber and subsequently flooded it with 1 bar of dry helium gas. The repetition rate of the few-cycle Tm-doped fiber laser was incrementally increased, which produced a proportional increase of the mid-IR output average power. At a maximum pump power of 31.4 W, which emerges from the ARHCF, we measured an average mid-IR power of 450 mW with a spectrum that spans from 7.3 µm to 16.5 µm (−10 dB intensity bandwidth) at a pulse repetition rate of 1.25 MHz. This result corresponds to a conversion efficiency of 1.8% assuming 25 W of average pump power in the GaSe crystal, which accounts for Fresnel reflection at the crystal surface and losses of steering optics after the ARHCF. The collimated mid-IR beam profile is depicted in Fig. 5c and reveals a slight beam deformation. We attribute this to the onset of thermal degradation of the collimating gold-coated parabola after the GaSe crystal, which prevented further increase of the pulse repetition rate. Assuming a nearly diffraction-limited beam quality, the brightness of this high-power mid-IR source has been calculated (refer to supplementary information) and compared with state-of-the-art results based on IPDFG pumped at a 1 µm wavelength. The result of IPDFG pumped at a 2 µm wavelength is depicted in Fig. 6 (PSD is provided in the supplementary information) and reveals a significant increase of brightness and bandwidth compared with IPDFG pumped at a 1 µm wavelength^27^, whereas the conversion efficiency of 1.8% is higher by nearly one order of magnitude. For comparison, the brightness of third-generation synchrotron radiation in this wavelength range^6^, which is also depicted in Fig. 6, is lower by several orders of magnitude, which substantiates the potential of this novel table-top mid-IR source for future applications. The presented front end based on a Tm:FCPA delivers a variable pulse train that is tunable in the pulse repetition rate, which closes the gap between conventional laser systems that are capable of delivering either low repetition rates in the range 100 Hz−1 kHz^21,22,26^ (low average power) or very high repetition rates in the range 10−100 MHz^19,27,28^ (low peak power). Thus, the system presented in this paper provides unique output parameters, which facilitates unprecedented sensitivity for background-free detection (e.g., electro-optical sampling^27^). This technique is based on nonlinear back conversion, which benefits from high peak power in the mid-IR and, in parallel, from a high photon flux.

## Conclusions

We have demonstrated a source of coherent mid-IR radiation with an unprecedented combination of average power, peak power, and spectral coverage in the molecular fingerprint region. At average power in excess of 150 mW, the spectral coverage can be tuned between 3.7 and 18 µm. The source is based on IPDFG, which is a nonlinear frequency downconversion process that affords the advantages of phase stability and simplicity^24–27^. This recent advancement in table-top Tm-doped fiber laser technology^30,34,36,37^ offers a flexible selection of laser parameters with respect to the targeted application in terms of pulse repetition rate and pulse energy (0.5 mJ/10 kHz to 0.5 µJ/100 MHz) as a starting point for mid-IR generation. The combination of GaSe as a nonlinear medium and 2-µm radiation for driving IPDFG, combined with ongoing further development of ultrafast 2-µm technology promises further power scalability of the scheme presented in this paper. The demonstrated power level renders this approach a truly viable table-top alternative to broadband synchrotron mid-IR radiation. In addition, IPDFG intrinsically ensures the formation of a CEP stable output pulse train^33^, which provides ideal prerequisites for spectroscopic and microscopic techniques based on the control and observation of the electric field of the mid-IR radiation^27^.

## Electronic supplementary material

Supplementary Material
