# Supplementary material for: Watt-scale super-octave mid-infrared intrapulse difference frequency generation
Source: Light Sci Appl. 2018 Nov 28;7:94. doi: 10.1038/s41377-018-0099-5 (PMC6258765; doi:10.1038/s41377-018-0099-5)
Supplement: Supplementary file 1 — Supplementary Material [file 41377_2018_99_MOESM1_ESM.docx]

SUPPLEMENTARY MATERIAL

Watt-scale super-octave mid-infrared intrapulse difference frequency generation

Christian Gaida^†1^, Martin Gebhardt^1,2^, Tobias Heuermann^1,2^, Fabian Stutzki^1,3^, Cesar Jauregui^1^, Jose Antonio-Lopez^4^, Axel Schülzgen^4^, Rodrigo Amezcua-Correa^4^, Andreas Tünnermann^1,2,3^, Ioachim Pupeza^5^, Jens Limpert^1,2,3^

^1^Institute of Applied Physics, Abbe Center of Photonics, Friedrich-Schiller-Universität Jena, Albert-Einstein-Str. 15, 07745 Jena, Germany;
^2^Helmholtz-Institute Jena, Fröbelstieg 3, 07743 Jena, Germany;
^3^Fraunhofer Institute for Applied Optics and Precision Engineering, Albert-Einstein-Str. 7, 07745 Jena, Germany;
^4^CREOL, College of Optics and Photonics, University of Central Florida, Orlando, Florida 32816, USA; ^5^Max-Planck-Institute of Quantum Optics, Hans-Kopfermann-Str. 1, 85748 Garching, Germany

†Correspondence: C Gaida, Email: [christian.gaida@uni-jena.de](mailto:christian.gaida@uni-jena.de)

**GaSe dispersion properties**

The dispersion of GaSe in the wavelength region of interest was estimated by the extended Sellmeier Equation with the coefficients shown in table S1 for the extraordinary and ordinary crystal axis^1^ :

$$n^{2}=A+\frac{B}{\lambda^{2}}+\frac{C}{\lambda^{4}}+\frac{D}{\lambda^{6}}+\frac{E}{1-\frac{F}{\lambda^{2}}}$$

|  | **A** | **B** | **C** | **D** | **E** | **F** |
| --- | --- | --- | --- | --- | --- | --- |
| ***n*_o_** | 7.443 | 0.405 | 0.0186 | 0.0061 | 3.1485 | 2194 |
| ***n*_e_** | 5.76 | 0.3879 | -0.2288 | 0.1223 | 1.855 | 1780 |

**Table S1.** Sellmeier coefficients of GaSe ^1^ for ordinary (*n*_o_) and extraordinary (*n*_e_) crystal axis.

**Estimation of mid-IR power**

Schematic setup of the DFG is shown in Fig. 3a of the manuscript. The generated mid-IR radiation passes several optical elements before it is measured by a thermal power meter (power *P*_M_) and spectrally characterized by a Fourier-transform interferometer (spectral power distribution S_M_). The corrected spectral power distribution after the GaSe crystal is estimated as follows:

$$S(\lambda)=\frac{S_{M}(\lambda)}{R_{\mathrm{ZnSe}}(\lambda)\cdot(1-R_{\mathrm{ZnSe}}\left( \lambda\right))^{2}\cdot R_{\mathrm{Gold}}^{2}\cdot T_{\mathrm{longpass}}(\lambda)}$$

The generated mid-IR after the uncoated GaSe crystal is reflected by 1 gold parabola, 1 uncoated ZnSe-wedge and 1 gold mirror and passes through 1 uncoated ZnSe-window and 1 longpass filter. The gold parabola and gold mirror have a reflectivity *R*_Gold_<97% in the mid-IR, each. The ZnSe-wedge reflection has been characterized at 2 µm wavelength and near 0 degree angle of incidence revealing a reflectivity of *R*_ZnSe_=17.6%. According to the dispersion of ZnSe ^2^, the Fresnel reflectivity decreases slightly with wavelength (see Fig. S1a) and matches the measured reflectivity at 2 µm very well. This also allows for an accurate estimation of the uncoated ZnSe-window transmission. Together with the transmission properties of the used longpass filter (see Fig. S1b)^3^, the measured spectral power distribution can be corrected accordingly. For wavelengths longer than 15 µm a transmission of >65% was assumed. The power P_M_, which was measured with a thermal power meter after all elements, is related to the measured spectrum *S*_M_. Thus, the total mid-IR average power after the GaSe crystal can be determined:

$$P=P_{M}\frac{S(\lambda)}{S_{M}(\lambda)}$$

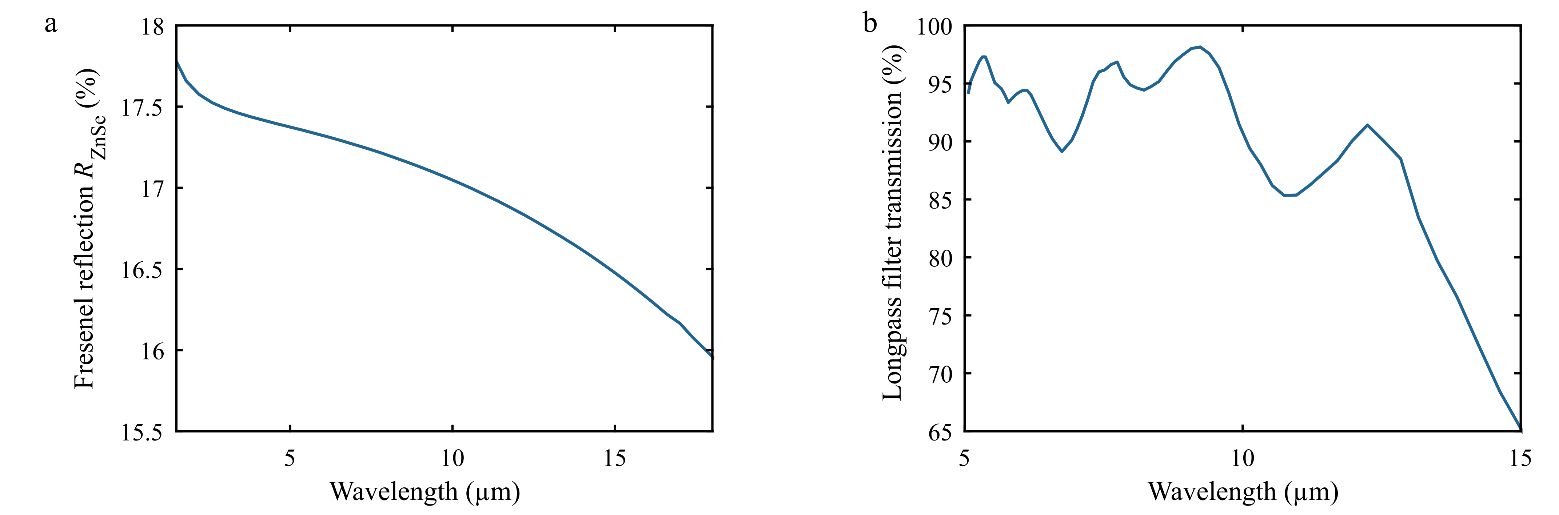


**Figure S1.** Wavelength-dependent reflection and transmission properties. **a** Fresnel reflection of ZnSe at normal angle of incidence. **b** Transmission of longpass filter to block the pump light at 2 µm wavelength ^3^.


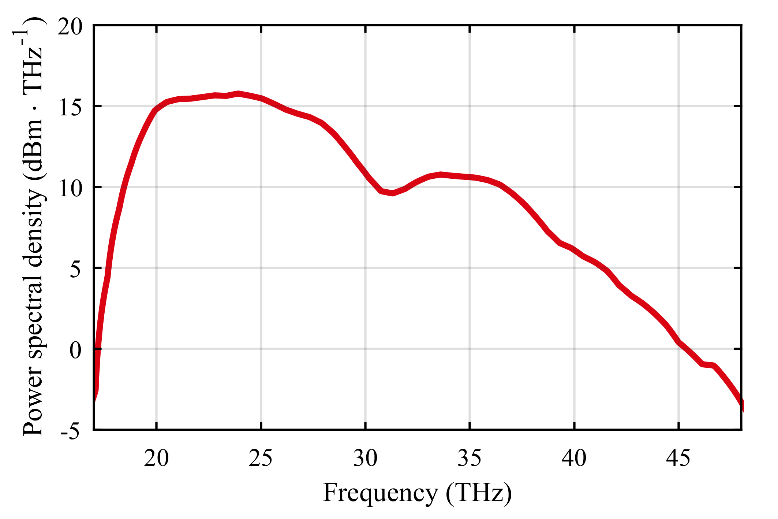


**Figure S2.** High-power mid-IR spectrum generated by intrapulse difference frequency generation, pumped at 2 µm wavelength resulting in a total mid-IR power of 450 mW.

**Brightness estimation**

The power spectral density (*PSD*) is used to calculate the power (Δ*P*) within a 0.1% interval of optical frequency *f* (Δ*f*):

$$\Delta P\left( f \right)=PSD\left( f \right)\cdot\Delta f$$

From this we can determine the photon flux ($\Phi$) within this optical frequency interval (Planck constant h):

$$\Phi(f)=\frac{\Delta P}{h\cdot f}$$

The brightness B can then be estimated as:

$$B\left( f \right)=\frac{\Phi\left( f \right)}{\pi\cdot w_{0}^{2}\cdot\Omega\left( f \right)}$$

Due to the spatial coherence of the DFG process we assume a diffraction-limited mid-IR beam that emerges from the focus (radius *w*_0_) in the crystal. Thus, the beam divergence in terms of solid angle Ω is given by (speed of light c):

$$\Omega(f)=\frac{c^{2}}{\pi\cdot w_{0}^{2}\cdot f^{2}}$$

Therefore, the calculation of the brightness can be simplified to^4^:

$$B\left( f \right)=\frac{\Phi\left( f \right)}{c^{2}}f^{2}$$

**References**

1 EKSMA. Infrared nonlinear crystals. <http://eksmaoptics.com>; 2017.

2 Marple DTF. Refractive Index of ZnSe, ZnTe, and CdTe. *Journal of Applied Physics* 1964; **35**: 539-542.

3 Edmund Opitcs. Longpass-Edge Filters, 4.5µm cut on filter. http://www.edmundoptics.com; 2017.

4 Shukla P, Lawrence J, Zhang Y. Understanding laser beam brightness: A review and new prospective in material processing. *Optics & Laser Technology* 2015; **75**: 40-51.
